# Effectiveness of a Teaching Session on Interpreting Common Orthopaedic Radiographs at a Major Trauma Centre

**DOI:** 10.7759/cureus.110678

**Published:** 2026-06-11

**Authors:** Hamza Ahmed, Aima Gilani, Marium Rizwan, Abdur Rehman, Mohamed Said Ammar, Muhammad Abdulvahab

**Affiliations:** 1 Trauma and Orthopaedics, Salford Royal NHS Foundation Trust, Salford, GBR; 2 Trauma and Orthopaedics and Spinal Surgery, Salford Royal NHS Foundation Trust, Salford, GBR

**Keywords:** fracture detection, junior doctors, orthopaedic x-ray, quality improvement, radiograph interpretation, trauma radiology

## Abstract

Background

Accurate interpretation of common orthopaedic radiographs is an important skill for junior clinicians working in emergency, trauma, and orthopaedic settings. Missed fractures and unrecognised malalignment remain important causes of diagnostic error, particularly in busy major trauma centres where the first review of imaging may take place before a formal radiology report is available. Structured, case-based teaching may improve both diagnostic accuracy and clinician confidence, but local educational interventions should be evaluated using objective measures.

Methods

We conducted a prospective single-centre educational quality improvement study at a UK major trauma centre between January 2025 and March 2026. Foundation doctors, core trainees, physician associates, clinical fellows, and other early-career clinicians rotating through trauma and orthopaedics or the emergency department were invited to attend a 45-minute senior-led teaching session on a systematic approach to common orthopaedic radiographs. Participants completed a 25-item image-based assessment and a 0-10 confidence rating scale immediately before and after the session. Those who consented to follow-up were also invited to complete a voluntary six-week retention assessment. The primary outcome was change in total radiograph interpretation score. Secondary outcomes included domain-specific accuracy, self-rated confidence, and the frequency of predefined critical errors.

Results

Forty-eight clinicians completed paired pre- and post-session assessments. The mean test score increased from 58.9% (SD 11.3) before teaching to 82.1% (SD 11.3) immediately after teaching, giving a mean paired improvement of 23.2 percentage points (95% CI: 20.8-25.6; p < 0.001; Cohen’s d_z = 2.83). Mean self-rated confidence increased from 4.5 ± 1.6 before teaching to 7.7 ± 1.8 after teaching. Improvements were seen across fracture detection, recognition of alignment and dislocation, hardware assessment, assessment of view adequacy, and escalation planning. Thirty-four participants completed the six-week retention assessment, with a retained mean score of 76.8% (SD 11.7). Critical errors fell from 146 before teaching to 48 after teaching, representing a 67.1% relative reduction.

Conclusions

A single structured, interactive orthopaedic X-ray teaching session was associated with significant immediate improvements in image interpretation accuracy and self-rated confidence among early-career clinicians at a major trauma centre, with partial retention at six weeks. Embedding short, recurring, case-based radiograph teaching into trauma and orthopaedic induction may be a practical way to support diagnostic safety. Further multicentre studies using validated image banks and patient-linked diagnostic outcomes are warranted.

## Introduction

Plain radiography remains the first-line imaging modality for most suspected appendicular skeletal injuries [1]. It is used throughout the patient pathway, from EDs and urgent care units to fracture clinics, inpatient orthopaedic wards, and perioperative trauma pathways. Although formal radiology reporting provides definitive interpretation in many settings, early clinical decisions are often made before those reports are available. In practice, junior doctors, emergency clinicians, physician associates, and orthopaedic trainees are frequently the first to review the images and decide whether immobilisation, further imaging, referral, or escalation is needed [2]. This is particularly relevant in major trauma centres, where high imaging volume, time-critical decisions, shift work, and frequent staff rotation make a reliable shared approach to X-ray interpretation essential.

Common orthopaedic radiographs can be more difficult to interpret than they first appear. Normal paediatric growth plates, subtle cortical breaches, non-displaced fractures, occult periprosthetic injury, malalignment, inadequate positioning, joint subluxation, and associated injuries can all be missed. These errors may lead to inappropriate discharge, delayed immobilisation, delayed operative referral, or unnecessary additional imaging [3]. The consequences are not limited to diagnostic delay. Patients may re-attend with ongoing pain, fractures may displace, theatre planning may be affected, and confidence in the service may be reduced. At the same time, over-calling normal variants as fractures can also cause harm, including unnecessary immobilisation, avoidable clinic referrals, further radiation exposure, and patient anxiety [4].

The need for better training in radiograph interpretation has been recognised for many years. Classic studies of junior doctors working in accident and EDs showed substantial rates of missed radiographic abnormalities and highlighted the particular risk of trauma X-ray misinterpretation during nights and weekends [2]. Guly reported that diagnostic errors in an ED were most often due to misreading radiographs, with many involving missed fractures [5]. More recent work has continued to show that skeletal radiography is an area in which junior clinicians often feel underprepared, despite how frequently they use it in front-line practice [6].

Radiology education for junior clinicians is often inconsistent. It may depend on local enthusiasm, clinical workload, and whether teaching opportunities arise during a particular rotation. Junior doctors have reported a preference for interactive, system-based, and case-based teaching rather than passive lecture-style sessions [7]. This is consistent with broader medical education evidence suggesting that active learning, deliberate practice, timely feedback, and clinically realistic cases are more likely to improve image interpretation than isolated lectures alone [8]. In orthopaedics, a structured approach is especially useful because accurate interpretation requires more than simply identifying a fracture. Clinicians must also judge image adequacy, assess alignment, recognise fracture patterns, evaluate implants, and understand the management implications.

At our major trauma centre, informal feedback from foundation doctors and clinical fellows suggested that many early-career clinicians felt underconfident when interpreting common orthopaedic X-rays at the start of trauma and orthopaedic rotations. Local governance discussions also identified variation in how X-ray findings and escalation plans were documented in emergency and ward settings. In response, we developed a concise, senior-led teaching session focused on the systematic interpretation of common orthopaedic radiographs encountered in everyday practice. The session used a reproducible checklist: confirm patient details and image adequacy; compare available views; assess alignment, bones, cartilage and joints, and soft tissues; identify any implants; state the diagnosis; and document the immediate management and escalation plan.

The aim of this study was to evaluate whether a single structured orthopaedic X-ray teaching session improved diagnostic accuracy and self-rated confidence among early-career clinicians at a major trauma centre. We hypothesised that participants would show a significant improvement in image-based assessment scores immediately after teaching, with partial retention at six weeks.

## Materials and methods

Study design and setting

This was a single-centre educational quality-improvement study conducted at a UK major trauma centre providing 24-hour emergency care, orthopaedic trauma, spinal, pelvic, and limb reconstruction services. The study took place between January 2025 and March 2026. Teaching sessions were delivered during departmental induction and repeated education sessions to capture clinicians rotating through trauma and orthopaedics or the ED over the course of the study period.

Participants

Eligible participants were clinicians expected to interpret, document, or act on plain orthopaedic radiographs as part of routine clinical practice. This included foundation doctors, core surgical trainees, clinical fellows, junior ED doctors, and physician associates. Radiology registrars and consultants, trauma and orthopaedic registrars at ST3 level or above, clinicians who had already attended the same teaching session during the study period, and participants with incomplete paired assessments were excluded. Attendance at the teaching session formed part of routine departmental education, and consent was obtained for anonymised assessment data to be used for service evaluation and quality-improvement purposes.

Teaching intervention

The intervention consisted of a 45-minute face-to-face interactive teaching session delivered by a trauma and orthopaedic consultant or senior registrar. Teaching used anonymised radiographs from the institutional teaching archive and focused on cases commonly encountered by early-career clinicians in emergency and trauma settings. These included distal radius fractures, suspected scaphoid injuries, elbow fat-pad signs, shoulder dislocations, neck of femur fractures, pelvic ring injuries, ankle fracture-dislocations, paediatric buckle fractures, tibial plateau fractures, periprosthetic fractures, post-operative fixation failure, and normal comparison films.

The session was designed to be practical and clinically focused rather than subspecialty exhaustive. A structured checklist approach was used throughout, with immediate discussion and feedback after each case. The practical checklist used during the teaching session is provided in the Appendix 1. The learning objectives were to apply a consistent ABCS-style approach (alignment, bones, cartilage/joints, and soft tissues) to orthopaedic radiographs; recognise common fractures and dislocations requiring urgent escalation; identify inadequate or missing radiographic views; avoid common normal-variant pitfalls; document interpretation and management plans clearly; and recognise when senior orthopaedic or radiology input was required.

Assessment instrument

Participants completed a 25-item image-based assessment immediately before and immediately after the teaching session. The pre- and post-session assessments used different but matched cases covering five domains: fracture detection, radiographic anatomy and orientation, dislocation and joint alignment, hardware and implant assessment, and initial management or escalation planning. Each item was scored as correct or incorrect using a predefined answer key agreed by two trauma and orthopaedic consultants and one musculoskeletal radiologist. Participants who consented to follow-up were invited to complete a voluntary six-week retention assessment using a third matched assessment set.

Self-rated confidence was recorded before and after teaching using a 0-10 confidence rating scale, where 0 meant “not confident at all” and 10 meant “fully confident to independently interpret common orthopaedic radiographs at my level and escalate appropriately.” Critical errors were defined before analysis as missed fracture, missed dislocation or subluxation, missed implant or fixation failure, inappropriate acceptance of inadequate imaging, or failure to escalate an urgent abnormality.

Outcome measures

The primary outcome was the paired change in total assessment score immediately before and after the teaching session. Secondary outcomes included changes in domain-specific accuracy, change in confidence score, six-week retention score among follow-up respondents, and change in the frequency of predefined critical errors. We also explored whether clinician grade was associated with baseline performance or magnitude of improvement.

Statistical analysis

Data were analysed using descriptive statistics. Continuous variables are presented as mean and SD when approximately normally distributed, and as median with interquartile range when distributions are skewed. Paired pre- and post-session scores were compared using paired t-tests, while Wilcoxon signed-rank tests were planned for markedly non-normal distributions. Categorical variables are presented as counts and percentages. The effect size for paired improvement was calculated using Cohen’s d_z, and statistical significance was defined as p < 0.05.

## Results

Participant characteristics

Forty-eight clinicians completed paired pre- and post-session assessments and were included in the primary analysis. Of these, 34/48 participants (70.8%) completed the optional six-week retention assessment. The participant group reflected clinicians who commonly provide first-line review of orthopaedic radiographs within emergency and trauma pathways. Foundation doctors formed the largest group, followed by clinical fellows and core trainees.

Primary outcome: total assessment score

The mean total assessment score improved from 58.9% ± 11.3% before the session to 82.1% ± 11.3% immediately after the session. This represented a mean paired improvement of 23.2 percentage points, with a 95% confidence interval of 20.8 to 25.6 percentage points. The improvement was statistically significant on paired-samples t-test, t(47) = 19.44, p < 0.001, and corresponded to a large within-participant effect size, Cohen’s d_z = 2.83.

**Figure 1:**
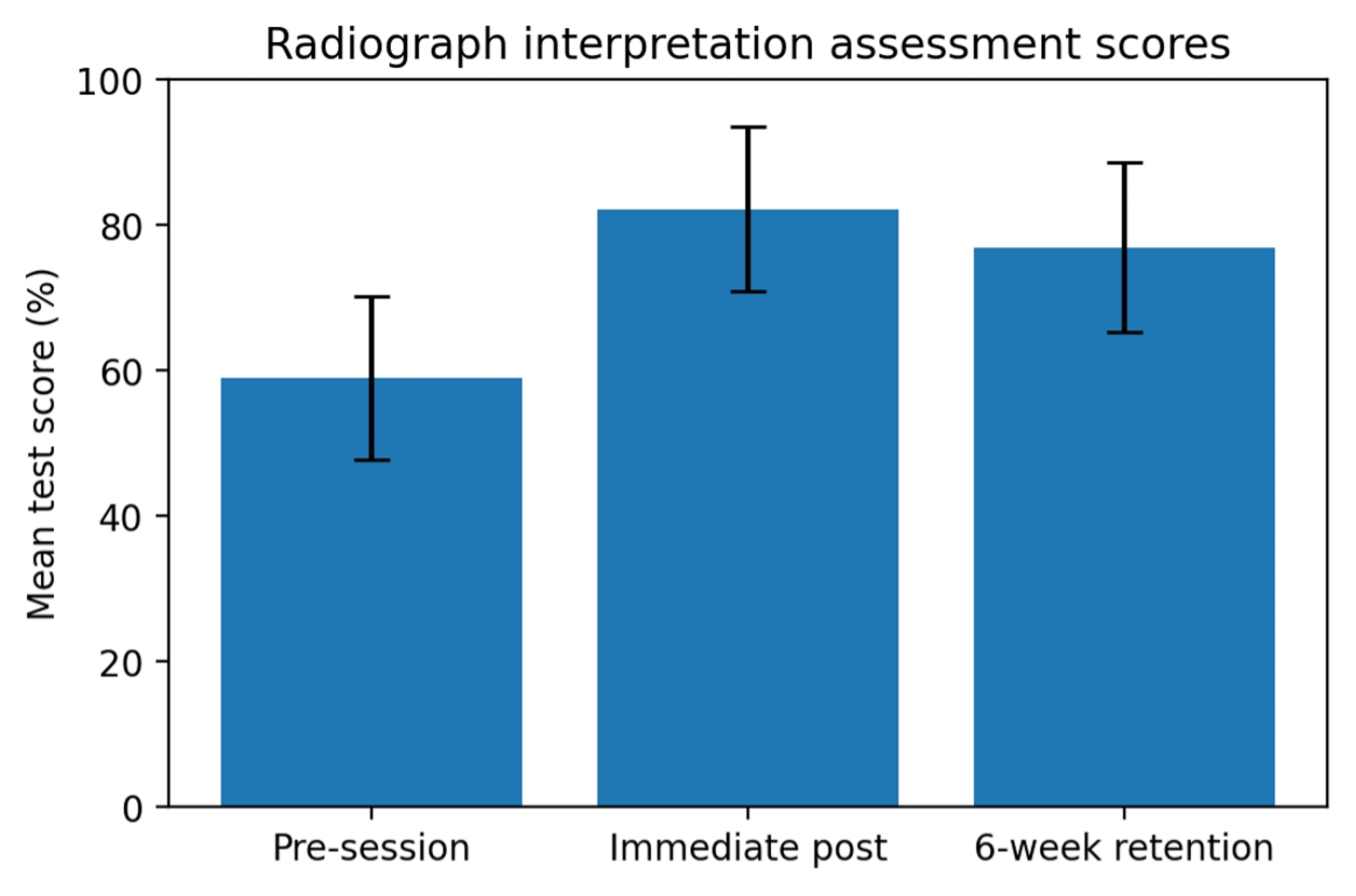
Mean radiograph interpretation assessment scores before teaching, immediately after teaching, and at six-week retention. Data are presented as mean ± SD. Error bars show SD. The pre-session and immediate post-session comparison included 48 paired participants and was analysed using a paired-samples t-test, t(47) = 19.44, p < 0.001. The six-week retention score is shown descriptively for the 34/48 participants (70.8%) who completed the optional retention assessment. Statistical significance was defined as p < 0.05.

Among the 34/48 participants (70.8%) who completed the six-week retention assessment, the mean score was 76.8% ± 11.7%. This was lower than the immediate post-session score among the same respondents but remained higher than the baseline score, suggesting partial retention of learning over time. The primary and secondary quantitative outcomes are summarised in Table 1, and mean assessment scores over time are shown in Figure 1.

**Table 1:** Primary and secondary quantitative outcomes. Data are presented as mean ± SD unless otherwise stated. Retention completion is presented as n/N (%). Mean change refers to the paired within-participant change from the pre-session assessment to the immediate post-session assessment. Test statistics are reported as t-values with degrees of freedom for paired-samples t-tests. Statistical significance was defined as p < 0.05. NA: Not applicable; pp: Percentage points.

Outcome	n	Pre-session mean ± SD	Post-session/retention mean ± SD	Mean change	95% CI	Test statistic	p-value	Statistical test
Total assessment score (%)	48	58.9 ± 11.3	82.1 ± 11.3	23.2 pp	20.8 to 25.6	t(47) = 19.44	<0.001	Paired-samples t-test
Confidence score (0-10)	48	4.5 ± 1.6	7.7 ± 1.8	3.2	2.9 to 3.5	t(47) = 21.47	<0.001	Paired-samples t-test
Six-week retention score (%)	34/48 (70.8%)	NA	76.8 ± 11.7	NA	NA	NA	NA	Descriptive only

Domain-specific performance

**Figure 2:**
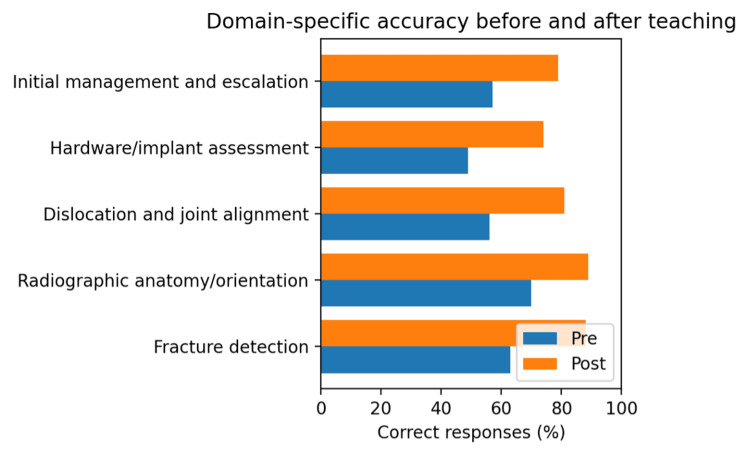
Domain-specific accuracy before and immediately after the teaching session. Data are presented as percentages of correct responses within each domain among 48 paired participants. Absolute improvements ranged from 19.0 to 25.0 percentage points across domains. Domain-level comparisons are shown descriptively; formal paired statistical testing was not performed because item-level paired response data were not available. Statistical significance for the overall assessment score is reported in Table 1.

Accuracy improved across all assessed domains. The largest gains were seen in hardware and implant assessment, as well as in recognising dislocation or malalignment. These were also topics that participants had informally identified as areas of lower confidence before the session. Fracture detection improved by 25.0 percentage points. The improvement in initial management and escalation was also important, as it suggested that participants were not only identifying abnormalities more accurately but were also better able to link their findings to appropriate and safer next steps. Domain-specific accuracy before and after teaching is summarised in Table 2 and Figure 2.

**Table 2:** Domain-specific accuracy before and after teaching. Data are presented as percentages of correct responses within each domain. Pre- and immediate post-session domain scores were based on 48 paired participants; six-week retention scores were based on the 34/48 participants (70.8%) who completed the retention assessment. Absolute change is reported in percentage points. Domain-level values are descriptive; formal paired statistical testing was not performed at the domain level because item-level paired response data were not available for each domain. Statistical significance for the overall assessment score is reported in Table 1.

Domain	Items, n	Pre-session correct, %	Immediate post-session correct, %	Absolute change, pp	Six-week retention correct, %
Fracture detection	10	63	88	25	82
Radiographic anatomy/orientation	5	70	89	19	84
Dislocation and joint alignment	4	56	81	25	75
Hardware/implant assessment	3	49	74	25	69
Initial management and escalation	3	57	79	22	73

Critical errors

**Figure 3:**
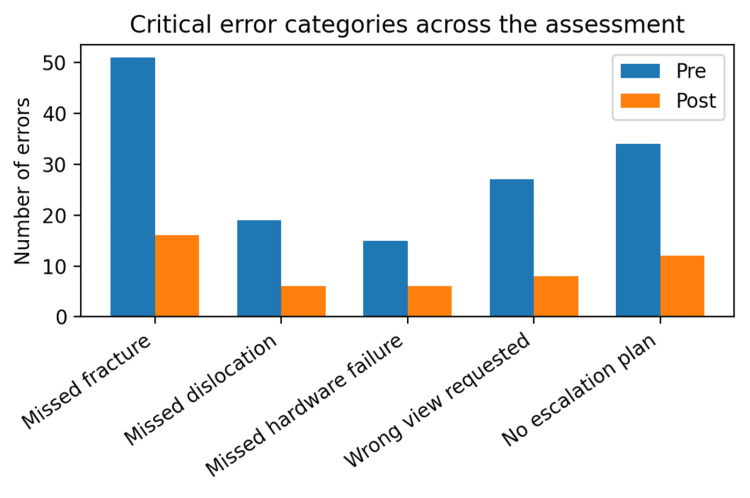
Critical error categories across the assessments before and after teaching. Data are presented as total counts of predefined critical errors across 48 paired assessments. Overall critical errors decreased from 146 before teaching to 48 after teaching, representing a 67.1% relative reduction. Category-level error reductions are shown descriptively; inferential testing was not performed because participant-level paired error data by category were not available.

Across the paired assessments, predefined critical errors fell from 146 before the session to 48 after the session, representing a 67.1% relative reduction. Missed fractures were the most common error before teaching and showed the largest numerical decrease after the session. Errors involving inadequate radiographic views and failure to document an appropriate escalation plan were also reduced substantially. Critical error categories before and after teaching are summarised in Table 3 and Figure 3.

**Table 3:** Critical error categories before and after teaching. Data are presented as counts of predefined critical errors identified across all paired assessments from 48 participants. Relative reduction was calculated as follows: ((pre-session errors - post-session errors) / pre-session errors) × 100. These data are descriptive event counts; inferential testing was not performed because participant-level paired error data by category were not available. Statistical significance for the overall assessment score is reported in Table 1.

Critical error category	Pre-session errors, n	Post-session errors, n	Absolute reduction, n	Relative reduction, %
Missed fracture	51	16	35	68.6
Missed dislocation or subluxation	19	6	13	68.4
Missed hardware or fixation failure	15	6	9	60
Inadequate or incorrect view accepted	27	8	19	70.4
No escalation plan for urgent abnormality	34	12	22	64.7
Total critical errors	146	48	98	67.1

Subgroup observations

Baseline scores varied by clinician grade. FY1 doctors had the lowest mean baseline score, while core trainees had the highest. However, all groups improved after the teaching session. The largest absolute improvement was seen among FY1 doctors, suggesting that the intervention addressed an important learning need at the point of transition into front-line trauma practice. The descriptive subgroup analysis by grade is presented in Table 4.

**Table 4:** Descriptive subgroup analysis by grade. Data are presented as n (%) and mean ± SD. Percentages were calculated using the total paired analysis cohort as the denominator (N = 48). Mean change is reported in percentage points. This subgroup analysis was descriptive and exploratory; no inferential comparisons between grades were performed. Statistical significance for the overall paired pre- to post-session improvement is reported in Table 1. FY1: Foundation Year 1; FY2: Foundation Year 2; CT1/CT2: Core Training Year 1/Core Training Year 2; pp: percentage points; n: number of participants; N: total sample size.

Grade	n (%)	Pre-session score, mean ± SD	Post-session score, mean ± SD	Mean change, pp
FY1	16/48 (33.3%)	52.8 ± 8.6	80.1 ± 9.5	27.3
FY2	14/48 (29.2%)	58.1 ± 9.2	81.6 ± 8.1	23.5
CT1/CT2	10/48 (20.8%)	66.4 ± 8.7	86.9 ± 7.0	20.5
Clinical fellow/locally employed doctor	8/48 (16.7%)	60.9 ± 10.1	81.3 ± 8.8	20.4
Total	48/48 (100.0%)	-	-	-

## Discussion

This single-centre educational quality-improvement study found that a structured 45-minute teaching session was associated with a significant immediate improvement in common orthopaedic X-ray interpretation among early-career clinicians at a major trauma centre. The improvement was large, occurred across several clinically relevant domains, and was accompanied by greater self-rated confidence and fewer critical errors. Although performance declined slightly by six weeks, scores remained above baseline, suggesting that the session had educational value but that reinforcement is likely to be needed over time.

These findings are consistent with earlier evidence showing that junior doctors may misinterpret clinically important trauma radiographs [2] and that diagnostic errors in emergency settings often involve missed fractures or radiographic misinterpretation [9]. More recent studies have also reported diagnostic uncertainty and variable confidence among foundation doctors interpreting fractures [10]. Taken together, this supports the need for practical, repeated, and clinically contextualised teaching, rather than assuming that confidence and competence in X-ray interpretation will develop passively during a clinical rotation.

A key strength of the intervention was its emphasis on a systematic approach rather than memorising isolated fracture appearances. The checklist encouraged participants to confirm image adequacy, review more than one view, assess alignment, bones, joints, soft tissues, and implants, and then state both the likely diagnosis and the required management or escalation. This reflects the cognitive structure used by more experienced clinicians and directly addresses several common sources of error. The reduction in errors related to absent escalation plans is particularly important. Safe practice does not depend on junior clinicians diagnosing every abnormality independently; it depends on recognising uncertainty, identifying concerning findings, and seeking senior or specialist input at the right time.

The improvement in hardware and implant assessment is also worth highlighting. Early-career clinicians often receive limited formal teaching on post-operative radiographs, yet major trauma centres frequently care for patients with plates, nails, arthroplasties, external fixators, and complex revision implants [11]. Recognising obvious malposition, periprosthetic fracture, fixation failure, or loss of reduction can be important during ward cover, emergency assessment, and out-of-hours decision-making. Including these cases in induction teaching may therefore reduce uncertainty and cognitive load during on-call shifts.

Our findings also fit with the broader radiology education literature. Junior doctors have previously expressed a preference for interactive, case-based, and system-based teaching [12], and systematic review evidence suggests that active learning approaches can improve radiology knowledge and skills [13]. Retention may be strengthened by a blended or repeated teaching model, as suggested by studies using e-learning and structured radiology resources [14]. In a major trauma centre, a practical model could include an initial face-to-face session, a short online case bank, monthly image review during trauma meetings, and formative reassessment midway through each rotation.

Limitations

This study has several limitations. The sample size was modest and drawn from a single centre, which limits generalisability. The assessment images were selected from a teaching archive and may not fully reflect real-time clinical practice, where interpretation is shaped by clinical history, examination findings, pain, time pressure, interruptions, and competing workload. The immediate post-session assessment may also partly reflect short-term recall, as it was completed directly after teaching. In addition, the six-week retention assessment was voluntary, creating a risk of response bias. Finally, the project measured assessment performance rather than patient-level outcomes, such as missed fracture rates, re-attendance, time to immobilisation, time to senior review, or delayed diagnosis.

Despite these limitations, the intervention is low-cost, feasible, and easy to reproduce. It requires a curated image bank, a clear answer key, senior clinical facilitation, and protected teaching time. The results support the inclusion of structured orthopaedic radiograph interpretation teaching in induction for clinicians working in emergency and trauma pathways. Future work should validate the assessment tool, involve multiple centres, compare face-to-face and blended delivery, assess longer-term retention, and link education to clinical safety outcomes such as discrepancy meeting findings, radiology addendum reports, delayed diagnoses, and incident reports.

## Conclusions

A structured, interactive teaching session on common orthopaedic X-ray interpretation was associated with improved assessment performance and greater confidence among early-career clinicians at a major trauma centre. It was also associated with fewer critical errors and partial retention of learning at six weeks. The intervention is practical to deliver during departmental induction and may support diagnostic safety when combined with regular case-based reinforcement, senior supervision, and radiology discrepancy feedback.

## Appendices

Appendix 1

**Table 5:** Practical checklist used during the teaching session.

Step	Prompt
1. Confirm context	Patient, side, date, mechanism, pain location, comparison films if available
2. Assess image adequacy	Correct views, entire bone/joint included, rotation and exposure acceptable
3. Alignment	Joint congruity, dislocation/subluxation, periprosthetic alignment
4. Bones	Cortical breach, trabecular disruption, avulsion fragments, subtle impacted fractures
5. Cartilage/joints	Joint space, intra-articular extension, effusion/fat-pad signs
6. Soft tissues	Swelling, gas, foreign body, lipohaemarthrosis
7. Hardware	Implant position, loosening, breakage, loss of reduction, periprosthetic fracture
8. Plan	Immobilisation, analgesia, weight-bearing advice, senior/radiology/orthopaedic escalation, documentation
